# Predictors of chronic kidney diseases and kidney failure in patients with CAKUT: a cohort study

**DOI:** 10.1007/s11255-026-05068-6

**Published:** 2026-03-24

**Authors:** Rosita Greco, Michele Provenzano, Agata Mollica, Giuseppe Pezzi, Michele Di Dio, Teresa Papalia

**Affiliations:** 1 Nephrology, Dialysis and Transplant Unit,’’SS. Annunziata’’Hospital, Cosenza, Italy; 2 Department of Medical and Surgical Sciences, University of Catanzaro, 88100 Catanzaro, Italy; 3 Division of Urology, Department of Surgery, Annunziata Hospital, Cosenza, Italy; 4 Department of Pharmacy, Health and Nutritional Sciences, University of Calabria, 87036 Rende, Italy

**Keywords:** CAKUT, Chronic kidney disease, Albuminuria, Pediatric nephrology

## Abstract

**Background and aims:**

Congenital anomalies of the kidney and urinary tract (CAKUT) are a group of disorders responsible for the majority of pediatric end-stage renal disease cases. The CAKUT group is phenotypically variable and can affect the kidney alone and/or the lower urinary tract. The aim of the study was to assess the risk factors for the onset of chronic kidney disease (CKD) and the progression to kidney failure (KF, i.e., the need for dialysis or kidney transplantation) in a cohort of patients with CAKUT.

**Method:**

We conducted a longitudinal study at the Nephrology, Dialysis, and Transplantation Unit of Annunziata Hospital of Cosenza. We enrolled consecutive patients with CAKUT from 2005 to 2020. Risk factors for CKD and KF were assessed via Cox regression.

**Results:**

We studied 87 CAKUT patients (33 females and 54 males). Proteinuria and hypertension were present in 32.2% and 24.1%, respectively, and a high prevalence of urinary tract infections (UTI 56.3%). CAKUT distribution was: 24 bilateral parenchymal abnormalities (BA), 38 renal parenchymal unilateral diseases (PUD), and 25 tubular unilateral diseases (TUD). Proteinuria was more prevalent in BA than in PUD or TUD (54.2% vs 15.8% and 36%, *p* = 0.006), whereas regular growth was less frequent in BA (33.3% vs 81.6% and 88%, *p* < 0.001). During a median study follow-up of 205 months, 30 CKD events and 14 KF were registered. BA, anemia, proteinuria, and regular growth independently predicted CKD, while anemia and BA were associated with KF.

**Conclusion:**

The long follow-up in these children with CAKUT allowed us to find the predictive risk factors for CKD and progression of KF. Early and appropriate treatment of these risk factors could improve the prognosis of renal disease.

## Introduction

CAKUT constitutes one of the most frequent birth defects and represents the most common cause of CKD in the first three decades of life. Bilateral renal hypoplasia and dysplasia with or without concomitant urinary tract malformations are present in more than 50% of children and adolescents commencing renal replacement therapy (RRT) [1–5]. The natural course of CAKUT is very heterogeneous [6, 7] and covers a wide range of renal and urinary tract anomalies with various degrees of severity, but a prospective long-term study of more than 40 years showed that the history of CAKUT significantly increases the risk for kidney failure and has long-term consequences [8, 9]. However, although prenatal diagnosis of CAKUT has been recognized as a prominent feature of such conditions for more than two decades, only limited data are as yet available on the risk factors related to the occurrence of CKD in these patients [10, 11] and little information is available about the long-term prognosis of patients with CAKUT as they advance into adult life. Understanding the rate of renal function impairment and predicting the long-term outcome are critical for accurate management of these patients, not only to refine the CKD risk stratification but also to guide clinical decisions. Today, we live in an era in which new therapeutic treatments can reduce the progression of CKD and modify the risk of cardiovascular morbidity and mortality. The importance of early and accurate identification of individuals with CKD is now more important than ever [12]. The aim of the study was to assess the risk factors for the onset of CKD and the progression to kidney failure (KF), i.e., the need for dialysis or kidney transplantation in a cohort of patients with CAKUT.

## Materials and methods

### Study design and procedures

This is a historical cohort study examining 87 consecutive patients referred to our non-dialysis CKD clinic from January 1st, 2005, to December 1st, 2020. Non-dialysis CKD clinic relates to an outpatient service located at the Nephrology, Dialysis, and Transplantation Unit of Annunziata Hospital of Cosenza and University of Calabria (Italy). The cohort was originally built to collect information about the diagnosis and prognosis of CAKUT in young CKD patients, since the first visit in Nephrology that is considered the baseline of the study. All procedures performed in this study were in accordance with the ethical standards of the institutional and national research committee and with the 1964 Helsinki Declaration and its later amendments or comparable ethical standards. The enrolled overall sample set provided written informed consent, signed by adult patients or by parents of children, that, as guarantor, is retained by the corresponding author. No research ethics approval was obtained, since these data were obtained from routine clinical activity.

CAKUT were diagnosed as bilateral abnormalities (BA): patients with bilateral renal parenchymal abnormalities (dysplasia or hypoplasia) or bilateral urinary collecting system abnormalities (bilateral vesicoureteral reflux VUR or double kidney district DKD; renal parenchymal unilateral diseases (PUD): patients with isolated and unilateral renal parenchymal abnormalities; **renal** tubular unilateral diseases (TUD): patients with isolated and unilateral urinary collecting system abnormalities (VUR, posterior urethral valves PUV, bladder dysfunction BD, pyeloureteral junction stenosis PJS, DKD, or anatomic stasis such as pyeloureteral duplicity, megaureter, urethrocele, ureterovesical junction obstruction UJO), or with migration and fusion abnormalities (ectopic kidney, horseshoe kidney, renal malrotation).

Other than the cause of CAKUT, nephrologists involved in the study collected (at first visit) several clinical, anamnestic, and demographic information such as: presence of anemia (Hb < 11 g/dL) and proteinuria (24 h proteinuria > 150 mg/day), regular or irregular growth, presence of arterial hypertension, concomitant history of enuresis, or urinary tract infections. The use of bladder catheterization was also reported. After baseline visit, patients were followed for the primary end points which were: 1) the development of CKD, namely eGFR reduction related to the age of patients; 2) KF, namely confirmed eGFR reduction below 15 mL/min or need for kidney replacement therapy. Patients lost to follow-up were right censored at the time of the last outpatient visit.

### Statistical analysis

Continuous variables were reported as either mean ± standard deviation (SD) or median and interquartile range (IQR) based on their distribution. Comparison among CAKUT categories (BA, PUD, TUD) was assessed by one-way ANOVA or Kruskall–Wallis test. Categorical variables were analyzed using Chi-square test. For survival analysis, we computed incidence rate for primary end points. Rates were reported as number of events/person-time and 95% confidence intervals (CIs) were calculated assuming a Poisson distribution. Cox proportional hazards models were run to compute the hazard ratio (HR) and 95% CI because the cause-specific relative hazards are more appropriate for studying the cause of diseases in the case of weak competing events. In fact, in our cohort, no death before CKD or KF was observed. The first model testing the predictors of CKD was adjusted for the cause of disease, anemia, proteinuria and regular growth. Predictors were identified a priori (knowledge-driven method) as potential risk factors based on previous literature [13, 14]. The second model tested the predictors of KF, including anemia, regular growth, and cause of CAKUT. In this case, the causes of CAKUT were grouped as bilateral vs. others (2 categories only) to avoid model overfitting given the low number of KF events. A two-tailed *p* value < 0.05 was considered significant for all analyses. Data were analyzed using STATA version 14 (Stata Corp. College Station, TX, USA).

## Results

We studied 87 patients with CAKUT, 33 females and 54 males (Table 1). The overall population was characterized by a moderate prevalence of proteinuria (32.2%) and hypertension (24.1%) and a high prevalence of urinary tract infections (UTI 56.3%).

**Table 1:** Basal characteristics of patients overall and by types of CAKUT (BA, bilateral abnormalities; PUD parenchymal unilateral diseases; TUD, tubular unilateral diseases)

	Overall (*n* = 87)	BA (*n* = 24)	PUD (*n* = 38)	TUD (*n* = 25)	*p*
Age, *months*	**3.0 [0.1–36.0]**	**0.1 [0–1.0]**	**6.0 [2.0–84.0]**	**3.0 [1.0–24.0]**	** < 0.001**
Male gender, *%*	**62.1**	**83.3**	**52.6**	**56.0**	**0.040**
Hypertension, *%*	24.1	37.5	15.8	24.0	0.151
Proteinuria, %	**32.2**	**54.2**	**15.8**	**36.0**	**0.006**
Anemia, *%*	**25.2**	**62.5**	**5.3**	**20.0**	** < 0.001**
Enuresis, *%*	28.7	45.8	18.4	28.0	0.067
Urinary tract infections*, %*	56.3	70.8	44.7	60.0	0.119
Bladder catheterizations, *%*	11.5	16.7	10.5	12.0	0.493
Regular growth, *%*	**70.1**	**33.3**	**81.6**	**88.0**	** < 0.001**

**Fig. 1:**
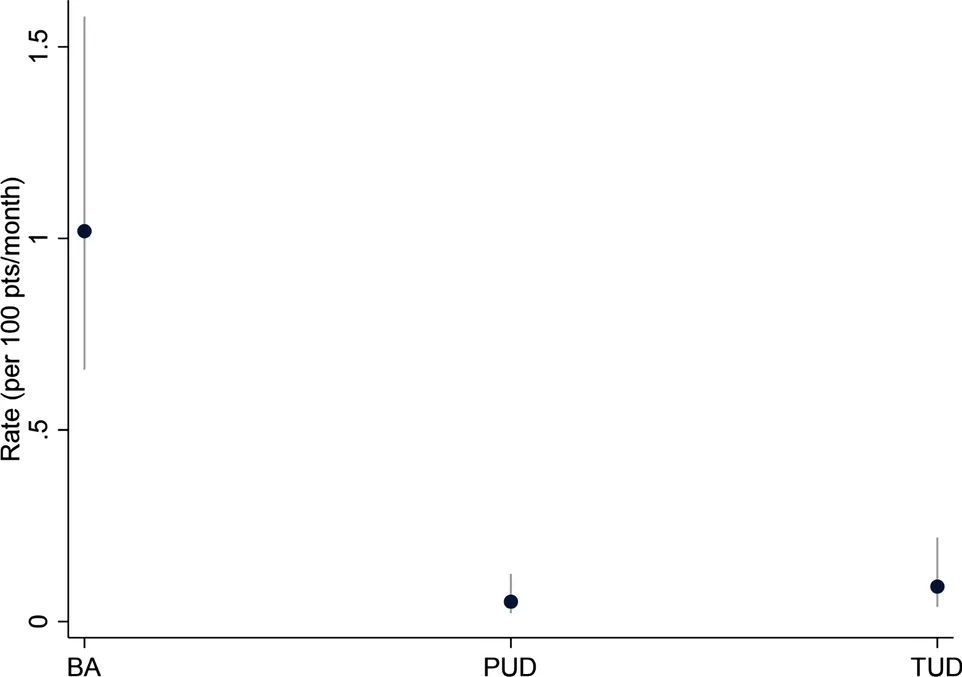
Incidence rate of chronic kidney disease according to CAKUT categories

CAKUT were distributed as follows: 24 were from BA, 38 from renal PUD, and 25 from TUD. Among CAKUT categories, bilateral diseases were more frequent in males (83.3%). Proteinuria was more prevalent in BA (54.2%) compared with PUD or TUD (15.8 and 36%, p = 0.006). Anemia showed a similar trend with a higher prevalence in the BA category (62.5%) versus PUD (5%) and TUD (20% with *p* < 0.001). Contrariwise, regular growth was less frequent in BA (33.3%) compared with PUD and TUD (81.6 and 88%, respectively, *p* < 0.001). Median study follow-up was 205 [IQR 88–288] months. Over this period, 30 CKD events and 14 KF were registered. The incidence rate of CKD was 1.02 (95% CI 0.66–1.58) per 100 pts/month in BA, 0.05 (95% CI 0.02–0.12) per 100 pts/month in PUD and 0.09 (95% CI 0.04–0.22) per 100 pts/month in TUD (Fig. 1).

The incidence rate of KF was higher in BA (0.42; 95% CI 0.23–0.76) than PUD (0.01; 95% CI 0.001–0.07) and TUD (0.03; 95% CI 0.001–0.14) per 100 pts/month. When testing the predictors of CKD (Table 2), anemia (hazard ratio HR, 2.9, p = 0.026) and proteinuria (HR 3.7, *p* = 0.008) were associated with a higher risk of CKD, whereas regular growth conferred protection against CKD (HR 0.18, *p* = 0.001). With respect to the cause of CAKUT, compared with parenchymal and tubular diseases, bilateral abnormalities were associated with an about fivefold increased risk of CKD (*p* = 0.010).

**Table 2:** Cox regression for the clinical predictors of chronic kidney disease development

	HR	95% CI	*p*
Anemia, *yes vs. no*	**2.90**	**1.14–7.41**	**0.026**
Proteinuria, *yes vs. no*	**3.66**	**1.40–9.56**	**0.008**
Regular growth, *yes vs. no*	**0.18**	**0.07–0.49**	**0.001**
Cause of CAKUT			
PUD	Ref	Ref	Ref
TUD	1.68	0.47–6.05	0.426
**BA**	**4.78**	**1.45–15.75**	**0.010**

*BA* bilateral abnormalities, *PUD* parenchymal unilateral diseases, *TUD* tubular unilateral diseases

With regard to KF, anemia (HR 29, p = 0.004) and bilateral cause of CAKUT (HR 33, p = 0.015) were found to be strong predictors (Table 3).

**Table 3:** Cox regression for the clinical predictors of kidney failure

	HR	95% CI	*p*
**Anemia, *yes vs. no***	**29.2**	**2.96–55.9**	**0.004**
Regular growth, *yes vs. no*	0.22	0.03–1.48	0.121
**Cause of CAKUT, *BA vs. other causes***	**32.9**	**1.97–54.98**	**0.015**

*BA* bilateral abnormalities, other causes encompass parenchymal unilateral diseases and tubular unilateral diseases

## Discussion

CAKUT comprise a large variety of malformations that arise from defective kidney or urinary tract development and frequently lead to KF. The CAKUT spectrum accounts for 20%–30% of congenital malformations and constitutes a frequent cause of birth defects [11, 15]. The clinical spectrum ranges from severe bilateral renal parenchymal or tubular malformations to isolated abnormalities [16, 17]. Understanding the rate of renal function impairment and predicting the long-term outcome are critical for accurate management of these patients. Limited information exists on the long-term natural course of CKD due to CAKUT, because in many children the molecular genetics is unknown and also because CAKUT comprise a heterogeneous rare disease group [18].

In this paper, we report longitudinal data on a cohort of 87 children referred to different renal and urinary tract anomalies from 2005 to 2020. The median study follow-up was 205 months. The mean age at the first visit was 3 months. We classified the heterogeneous rare disease group into three categories: bilateral abnormalities, unilateral renal parenchymal diseases, and unilateral tubular diseases. CAKUT were more prevalent in males than females (54 vs 33). This higher prevalence in males could be attributed to anatomical differences and nephrogenesis factors in the urinary tract [19]. Such a gender difference had also been observed in our children with bilateral CAKUT (83% males) compared with PUD (52.6%) and TUD (56%) (*p* = 0.040, Table 1). The relationship among the three CAKUT categories and the onset of CKD and progression to KF (i.e., the need of dialysis or kidney transplantation) has been studied. The incidence rate of CKD and KF was higher in BA than in PUD or TUD, associated with an about fivefold increased risk of CKD (*p* = 0.010, Table 1). Therefore, kidney survival appears to depend on the severity of renal dysplasia. As already demonstrated, children with severe dysplastic kidney disease require renal replacement therapies from early infancy; those with less severe malformations characteristically undergo a transient period of stable or even increasing kidney function resulting from compensatory hypertrophy of the remnant functioning nephrons [6, 18–21]. This research has also identified important differences in the long-term phenotypic evolution of different subgroups of CAKUT. Some children who belong to BA with severe renal dysplasia had CKD already in the first few months of life and showed early progression to KF.

We then analyzed the correlation between risk factors for CKD progression (hypertension, anemia, proteinuria, UTI, regular growth) and different CAKUT phenotypes in this pediatric population. The overall population was characterized by moderate prevalence of proteinuria (32.2%) and hypertension (24.1%) and high prevalence of UTI (56.3%). In previous studies, hypertension has been proven to be an independent risk factor for faster decline of glomerular filtration rate in renal patients, but long-term studies observing the progression of nephropathy in children with CAKUT are scarce [18, 20–22]. Our overall population was characterized by a moderate prevalence of hypertension, but there was no significant difference between the three groups (*p*. 0.151, tab1). These findings could be explained by careful outpatient nephrological monitoring and the recommendation for controlled salt intake. A high prevalence of UTI is related to intrinsic urinary anatomical abnormalities in the population examined and is not correlated with disease severity (*p* 0.119).

Anemia (hazard ratio HR, 2.9 *p* = 0.026) and proteinuria (HR 3.7, *p* = 0.008) were associated with a higher risk of CKD, whereas regular growth conferred protection against CKD (HR 0.18, *p* = 0.001). With respect to the cause of CAKUT, compared with parenchymal and tubular diseases, bilateral abnormalities were associated with an about fivefold increased risk of CKD (*p* = 0.010). Anemia and proteinuria are probably related to the lower nephron number or impaired nephron function due to alterations during nephrogenesis, identified as an indicator of altered kidney development [23, 24] and at a high risk of stage 2–5 CKD in CAKUT patients. This is more evident in bilateral diseases, as demonstrated in our work. Therefore, careful management of anemia and early use of antiproteinuric drugs can slow CKD progression. Our results show that regular growth is associated with better renal prognosis. This condition may depend on the absence of CKD features (anemia, metabolic acidosis, electrolyte anomalies, mineral-bone disorders, sexual hormone dysregulation, and malnutrition) in milder forms, even within BA. Thus, close monitoring during outpatient visits of regular growth, adequate nutrition, early treatment of metabolic acidosis and calcium/phosphorus axis dysfunction, and treatment of anemia and proteinuria could improve kidney survival.

Anemic patients with bilateral disease were at higher risk of KF. However, growth was not significantly associated with KF, probably because these children received specific treatments such as oral bicarbonate, growth hormone, vitamin D, and an age-normalized protein diet. The main key messages of the work are reported in Table 4.

**Table 4:** Principal evidence of the present study

CAKUT need to be sub-classified due to the clinical variability and phenotype.
Three CAKUT categories (bilateral abnormalities; parenchymal unilateral diseases; tubular unilateral diseases) are differently associated with risk of CKD (being higher in bilateral abnormalities then others) and progression to KF (being higher in bilateral abnormalities then others).
Regardless of the cause of CAKUT, traditional risk factors for CKD (anemia, proteinuria, regular growth) play a significant prognostic role in pediatric population
Try to outline a diagnostic and therapeutic pathway for patients with CAKUT with an early intervention on risk factors for KF.

*BA* bilateral abnormalities, other causes encompass parenchymal unilateral diseases and tubular unilateral diseases

In conclusion, bilateral abnormalities are decisive for clinical outcome, and although pediatric CKD is a relatively rare disease, its sequelae have a lifelong impact. Our findings underscore the importance of early diagnosis and a correct classification of CAKUT, because the cause displays an association with kidney outcomes. Moreover, regardless of the cause of CAKUT, several other factors are predictors of CKD or KF. A more appropriate disease classification, ideally combined with lifelong patient follow-up, will contribute to improving personalized disease management among patients with congenital kidney malformations. The early detection of risk factors for CKD and KF in CAKUT patients may allow improving their management as well. A long-term follow-up surveillance is needed to optimize renal prognosis and improve quality of life in this vulnerable population.

## Data Availability

No datasets were generated or analyzed during the current study.
